# Coming back from the edge: a qualitative study of a professional support unit for junior doctors

**DOI:** 10.1186/s12909-017-0978-0

**Published:** 2017-08-23

**Authors:** Elaine Wainwright, Fiona Fox, Tailte Breffni, Gordon Taylor, Michael O’Connor

**Affiliations:** 1 Psychology Department, Bath Spa University, Bath, UK; 2 Department for Health, University of Bath, Bath, UK; 3 National Institute for Health Research Collaboration for Leadership in Applied Health Research and Care West (NIHR CLAHRC West) at University Hospital Bristol NHS Foundation Trust, Bristol, UK; 4 Severn Postgraduate Medical Education (Deanery), Bristol, UK; 5 Department for Health, University of Bath, Bath, UK

**Keywords:** Qualitative research, Junior doctors, Work stress, Occupational support

## Abstract

**Background:**

It is known that many trainee doctors around the world experience work satisfaction but also considerable work stress in the training period. Such stress seems to be linked to multiple factors including workload, level of support and growing cultural inculcation into unwillingness to show any personal or professional weakness. In the United Kingdom, junior doctors are qualified medical practitioners who have gained a degree in Medicine and are now working while training to become a specialist (consultant) or a general practitioner. The period of medical training can be particularly stressful for some UK junior doctors, in common with their counterparts in other countries. UK Postgraduate Medical Deaneries provide support for those who need it via Professional Support Units (PSUs); however little is known about the perceptions and experiences of the doctors who access and utilise this support. This study aimed to generate qualitative insight into how the (PSU) provided by one UK Deanery is experienced by the trainees who accessed it. We aimed to investigate whether such experience intersects with the progressive socialisation of trainee doctors into the notion that doctors do not get ill.

**Methods:**

Through in-depth telephone interviews with eight female junior doctors, we explored the benefits and problems associated with using a PSU with reference to the formation of trainee doctors’ professional identities, and conducted a thematic analysis.

**Results:**

Themes identified illustrate the process of accepting, accessing and benefiting from PSU support. These are: Medical identity intact (it will never happen to me); Denial of disrupted medical identity; Being on the edge: accepting help; Role of PSU in ‘recovery’ process; Repaired identity / coming back from the edge; Different ways to be a doctor. The gendered sample occurred simply as it was females who responded to study invitations. Whilst we present some related aspects (such as “manning up” as part of keeping going), analyses of this small sample showed that medical identity as a doctor in training was more salient than a gendered experience of help seeking in this study.

**Conclusions:**

This study highlights the initial reluctance of female junior doctors to seek help from the PSU, as acknowledging their own difficulties spoiled their identity as a competent doctor. However, once engaged with the PSU, the findings exemplify its role in repairing medical identity, by offering different and acceptable ways to be a doctor. We interpret these findings within Goffman’s theoretical framework of stigma conferring a spoiled identity on recipients, and how this may then be repaired. Reducing the stigma attached to initial help-seeking among junior doctors is crucial to increase ease of access to the PSU and to improve the experiences of doctors who encounter challenges during their training.

## Background

It is well established that doctors can experience stress and burnout, adversely affecting job satisfaction and patient care [1–4]. Globally, junior doctors experience positive growth but also considerable stress, anxiety and even work attrition, as they progress from trainee to independent professional [5–7]. It has been reported that over half of UK medical trainees experienced personal illness, which was associated with higher anxiety and lower preparedness [8]; similar associations were found between illness and academic vulnerability in American trainees [9]. An Australian study of junior doctors found that 71% were concerned about their own health and met criteria for low job satisfaction [10]. A UK study of first-year junior doctors found that the transition from student to practising doctor was stressful, particularly regarding managing uncertainty, patient deaths and feeling unsupported [6]. Another UK study of trainee doctors in all years of training found that junior doctors tended to ‘burn out’ when more intense work stress led to perceptions of lower support [11]. Research also identifies difficulties for junior doctors in accessing sufficient informal social support, as perceived by their seniors [12], feeling inhibited from seeking help for one’s own ill health [13] and finding traineeships exceptionally stressful [14]. Literature indicates that doctors struggle to access timely help for physical, psychological and workplace issues across their whole careers; from medical student [9] to junior doctor [15, 16] and right through to senior doctor [17]. This may be related to the progressive socialisation into a cultural invulnerability to illness. For example, it has been found that junior doctors are concerned about disclosing significant personal illness for fear of being stigmatised by colleagues due to perceptions of an unspoken norm that doctors do not get ill, as this impacts negatively on patient care and colleagues’ workloads [13]. It has also been found that self-management of various psychological and physical illnesses is learnt early on in trainees’ careers and increases as they gain clinical access [18].

Within the UK National Health Service (NHS), Deaneries are regional academic organisations providing postgraduate medical education and training. Many Deaneries provide systems designed to prevent problems, including inductions, medical textbooks on smartphones [15] and buddy schemes [19]. Deanery Professional Support Units or PSUs [20] offer an educational assessment of problems with potential onward referral to counselling, coaching and other one-to-one resources. Despite this, many junior doctors continue to experience significant stressors at work [5]. NHS Digital data indicates that in 2015-16, 120 junior doctors left for reasons attributable to stress, e.g. “work life balance” or “health” [21]. Losing junior doctors incurs great financial and societal costs.

A recent review found that junior doctors perceive there is a lack of organisational support for difficulties encountered during training and recommended that further work should examine the potential benefits of different kinds of organisational support [7]. In addition, it is known that poor organisational culture, such as that which allows bullying, also impacts on junior doctors’ stress [22] and help-seeking behaviours. A recent study found that almost half of UK trainee doctors surveyed had experienced cyber-bullying which was significantly related to ill-health and job dissatisfaction [23]. The authors recommended that new policies and training (e.g. about appropriate use of emails and social media) are needed to mitigate these effects. A recent report found that whilst UK junior doctors often felt overburdened by factors relating to organisational structure (such as rotas suddenly changing), they also felt unable to seek help due to the stigma that might bring [24].

There is little literature which explores the transition from graduate student to professional, in-depth from students’ perspectives [15]. The demands of medical training, and trainees’ reluctance to seek help, have implications for the management of trainees. Not all trainees in need will access Deanery PSUs. However, given the relatively recent development of such services [20], and the sparse literature exploring trainees’ experiences of accessing PSUs [25] it is important to gain a better understanding of their role in supporting junior doctors who need help. Such research can also provide learning for the development of support services in different settings. We undertook an exploratory, in-depth qualitative study in order to explore the experiences of trainees who used the PSU in one UK Deanery. This study was conducted in partnership with the Deanery as a part of the evaluation of their PSU.

### Study setting

The PSU in this study promotes trainee well-being and personal development by providing support to tackle obstacles to performance, such as health concerns, psychological factors and environmental concerns [26]. PSU support entails an initial meeting with a non-medical case manager with a background in a relevant area, typically occupational psychology, counselling or human resources. Trainees may be referred by a more senior member of their team, or may self-refer. The typical journey is an initial meeting exploring concerns and constructing an action plan with the trainee, and subsequent follow-up. The action plan may sometimes drawing upon other external sources of support such as study skills tutors or counselling providers. In the majority of cases, individuals are seen by a PSU Case Manager for two meetings. They will have had additional meetings with other services the PSU has triaged them to, for example, counselling normally involves a package of 6 sessions and study skills, a package of 2-3 sessions. The number of sessions accessed is often determined by the complexity of the issues the individual is facing.

### Aim

The study aimed to explore junior doctors’ experiences of using a Deanery PSU and their perceptions of the impact it has on their career as they are forming their professional identities.

## Methods

The research team chose an in-depth qualitative study design to investigate the experiences of trainees using a PSU. Thematic analysis was considered appropriate as it is a flexible research tool, which can provide a rich, detailed account of data that is both descriptive and interpretive [27]. Thematic Analysis covers a range of epistemological and ontological decisions and was used as a ‘contextualist’ method within a critical realist paradigm [28]. This approach acknowledges ways in which people make sense of their experiences as well as how broader social structures impact on these, whilst remaining focused on the limits of the material [29]. Therefore it facilitated an over-arching understanding of the issues and meanings ascribed to using a support service for junior doctors.

### Participants and recruitment

While definitions of ‘medical trainee’ vary [6] this study included junior doctors from the first postgraduate year (Foundation) up to the year below consultant status. A sample of junior doctors, who had used the PSU in one UK Deanery, was recruited. The study was funded by the Deanery, who wanted to gain an in-depth understanding of service users’ experiences. Three administrative Deanery staff sent a generic email with brief study information to trainees who had accessed the PSU, and who had previously given permission to be contacted. Within a six month recruitment period, 20 people were contacted as this was the number who had completed service access in that time. Those who expressed an interest in participating (*N* = 10) were put in touch with a University researcher, who explained the study, answered queries, and enabled informed consent from 8 participants, who were all subsequently interviewed. Thus the response rate was 50% with regard to the initial email and 40% of contacted individuals were actually interviewed. There was no financial incentive offered to participants although they were offered one free hour of coaching if they wished. Recruitment was guided by the number of trainees who had used this relatively new service and who were willing to participate. Although small, this number was deemed reasonable for the exploratory qualitative design and to meet the study aims. All participants were female, compared to 65% (259/397) of PSU users overall and the participants were at various stages of training (see Table 1). We can only speculate about the reasons for the gendered response to our study invitation. It has been previously found that offering incentives means that women in the general population complete more items in telephone surveys than men [30], although we have not found any evidence that female doctors respond more than their male counterparts to requests to participate in interviews for research. It may be that the gendered response we encountered reflects the general social trend that more women than men seek help for psychological distress [31] and may be more willing to discuss their experiences. Although unplanned, the sample provided a valuable opportunity to explore the experience of female trainees.

**Table 1:** Participants’ training roles

Grade of junior doctor	Number of participants in study
Foundation Year One (F1)	1
Foundation Year Two (F2)	
Speciality/Core/Vocational years one to 8 depending on specialty (ST1/CT1/GPST1)	ST1 (1) ST3 (2) CT3 (1) ST4 (2) ST7 (1)

### Data collection and analysis

An experienced qualitative researcher (EW) conducted semi-structured telephone interviews between April 2014 – March 2015. Interviews lasted 35-100 min (mean: 50). The research team developed the interview schedule, with open questions, as well as more focused practical questions such as usability of the PSU website. Interviews were audio-recorded then transcribed verbatim.

Two researchers (EW and FF) with expertise in qualitative data analysis undertook the analysis, using NVivo software. They immersed themselves in the data by repeatedly listening to the recordings and reading verbatim transcripts. They generated initial codes and then considered how different codes may combine to form broader, more analytic themes. EW coded all data, and FF independently coded half the data. Together they debated and refined the analysis of all data (e.g. removing themes if not enough data supported them, or collapsing related themes into parent themes), until reaching thematic consensus. EW and FF were employed by two universities and were independent of the Deanery. Neither had any previous relationship with the study participants. The researchers attempted to be reflexive throughout, by (1) acknowledging their influence on shaping the data collection and analysis (2) coding data without fitting them to analytic preconceptions, so that the themes identified were strongly linked to the data themselves (3) offering participants the opportunity to view and comment on coding. Those who did so (two) did not suggest any changes (4) discussing coding regularly between EW and FF as well as with the wider research team.

## Results

The themes identified illustrate the process of accepting, accessing and benefiting from PSU support. These are: Medical identity intact (it will never happen to me); Denial of disrupted medical identity; Being on the edge: accepting help; Role of PSU in ‘recovery’ process; Repaired identity/coming back from the edge; Different ways to be a doctor. Shorter quotations are embedded within the text; for longer quotations see Table 2.

**Table 2:** Illustrative quotations for themes and sub-themes

Theme or sub-theme	Illustrative quotations
Medical identity intact/It will never happen to me	*“I think there is a huge psychological barrier, well, I found there was, in terms of admitting you are struggling with things, and again it might just be me personally, but there’s a certain amount of pride involved in not wanting to admit that you are struggling and not wanting to accept what’s going on”* (PPT7) “*It’s hard for a doctor to say. They’re used to helping people, so actually it’s really hard to say I might need some help”* (PPT 2)
Denial of disrupted medical identity	*“And I remember them saying so flippantly, almost as a tick-box exercise,” “You don’t need to see a counsellor, do you?” And actually, I really did … If I had had the support …right at the beginning, this hell is going to be prevented (PPT 8)* *“And also it’s very much a culture within the NHS that you are at work, because if you are not at work it means somebody else has double the work…If you don’t do it, it means your colleague has to do it and nobody likes to be in that position. Nobody likes to put their colleague in that position.”* (PPT3)
Being on the edge: accepting help	*“I felt a small amount of resentment or something, or the inference that I was a problematic… I know I was causing them headaches because I was stressed and causing issues … The inference is that you were incompetent or that you were… Do you know what I mean? That you would cause a problem clinically, that you were unsafe or. And so I felt a little bit resentful of that implication from the website even I knew that I wasn’t the case”* (PPT 5). *“I think it’s difficult to accept that sometimes you can be a patient and you can have something happen that means that you need extra help, and you don’t want to be seen by your colleagues or your managers as someone who can’t cope because you’re meant to be able to cope in emergency and difficult situations”* (PPT7) *“The other thing that I was a bit conscious about is when you… When I went for the meeting with the PSU. It’s done up in the Deanery House … there are quite a few people who are based up there from [speciality]… And so I… And there were all these like formal like annual reviews going on that are held there at different times of year for training and so I was also quite conscious of like well what am I going to say if I bump into someone I know?”* (PPT5)
Role of PSU in ‘recovery’ process/Making sense of their story	*“I felt that I was being supported and not judged, and they made that very clear. I found it really helpful…because until then, you felt that you were the only that was going through a difficult time and that was a failure on your part, and that was part of where I was mental state as well, thinking that everything that was negative against, it was all your fault and everyone else was fine” (PPT2)* *“I remember her (PSU staff member) saying to me,” “I can’t believe you’ve dealt with this all by yourself up until this point.” And I think actually just seeing someone almost visibly shocked by what I was telling her, just made me realise that no I’m not being pathetic … “Gosh, she almost can’t believe what I’m telling her.” And it was just the first time that I had actually thought, “Okay, this isn’t me just falling apart and losing it, because I’m pathetic, it’s actually because I’ve been through quite a difficult situation.” “So that was another pivotal moment I think”* (PPT 8)
Role of PSU in ‘recovery’ process: Focussing on their own health	*“I was off work and it’s the first time I’ve ever been off work, and you’re constantly feeling bad about it and you don’t know what everyone thinks, and you don’t know how long you should be off. And it’s really difficult and I think [PSU staff member] was very reassuring and saying,” “It sounds like the right thing you’re off, and do not rush back,” and you know, to kind of say it’s okay that you’re off work. “So that’s what I think was a really important thing”* (PPT 6)
Role of PSU in ‘recovery’ process: Understanding that they are not alone	*“So to go there and realise that of course you weren’t the only one, and many people have been here you are and have now gone on and are living and doing what they want to do, and not restricted by that. Just making you feel that it was the norm, because certainly as medics you kind of feel that that shouldn’t happen to you, which is ridiculous, but that’s how you feel. It’s [the NHS] very much a culture of man up and just get on with things. It [the PSU] felt the opposite of that attitude. It felt that actually you are a human, just like everyone else, this can happen, and here we are to support you, this is what we can do, and we want you to be the best that you can be. And so it was really encouraging and supportive, I felt”* (PPT2)
Repaired identity/Coming back from the edge	*“I mean they very much have your best interests at heart and really don’t want to lose you, you know. Kept making a big thing about you being, you know, how the Deanery wouldn’t want to lose you from the training programme and that it wasn’t going to be detrimental, and really they just want to try and help you get back on track really”.* (PPT 6)
Different ways to be a doctor	*“I think he fully understood how I was feeling [about on-calls] and he actually thought about cover there and then, and so immediately I just felt better because that was one thing I had actually been really worried about” (PPT1).* *“support helped in that the attitude was helpful in that some people do it this way, other people do it that way and that’s acceptable” PPT2).*
Breaking the cycle	*“that they stay in contact even after things have got better is a real positive because if you feel that things are even starting to head down the same sort of road again then you don’t feel like you’re going backwards because you’re already … you’re still in touch with them, so even though nothing is said in these emails you still feel like you’re in the system somehow”* (PPT 1) *“It’s people who have been there done that who end up being mentors to help other people. That’s really encouraging, particularly if there’s a risk of feeling like you’re the only one. But to see someone who is on the other side is much better, it’s really helpful*” (PPT 2) *“I just know if I was a registrar listening to all the things that I’ve had to go through I would have learnt so much and I wish I had known all of this before I went through this”* (PPT 8).

### Medical identity intact (It will never happen to me)

The participants initially assumed that they would never need PSU support and therefore did not prioritise information about it during their induction. Many could not recall hearing about it, although some admitted that “*you kind of hear about it … but you don’t remember*” (PPT 6). This can be explained by the prevailing medical identity which dictates that doctors should not show signs of personal weakness or take sick-leave. As one participant said, “*It’s [the NHS] very much a culture of man up and just get on with things*” (PPT 2). Used by a female trainee, the phrase “man up” was striking, perhaps consistent with masculine expectations of hiding weakness. However, all other participants, and even Participant 2 later in her interview, discussed the making, breaking and repair of their medical identities in terms of being “human” *(“you are a human, just like everyone else, this can happen”)* (PPT 2). None of the participants explicitly discussed their gender within the context of help seeking during the interviews. Participants identified that the PSU only became relevant when they encountered personal or professional problems and not before, as they had all started their traineeships with the expectation that taking time off is unacceptable. Participant 3 exemplified this in her comments that “*it’s kind of instilled in us from the start of our NHS life that you don’t create extra work for people [by being off work]”.*

### Denial of disrupted medical identity

All participants encountered personal and professional difficulties which impacted on their own health, wellbeing and work lives. However, they self-reported a tendency to *“bottle things up and trying to carry on”* (PPT 8) rather than to seek help. This reflected attempts to preserve their medical identity, and the lack of cultural fit between admitting the need for help and the stoicism required of doctors. Some participants seemed to self-stigmatise. For example participant 7 discussed how *“you don’t want to be seen by your colleagues or managers as someone who can’t cope”.* Other participants who did disclose their need for support, felt that even their opening disclosure was negatively received by managers, leading to further disruption of their self-concept as a competent doctor. For example, participant 5, who approached her clinical programme director about her need for a career break to rest and repair, reported her perception that he had “an agenda to it which was basically to try and stop me from having the break”. She explained that she only secured her break when she herself involved Occupational Health, having been referred to them by the PSU. Other participants reported that managers were supportive of their plans to access the PSU but they still felt stigmatised, as this did not align with their concepts of being a good doctor. Being marked out as ‘different’ is a hallmark of stigma, illustrated by participant 4, who reported that *“the whole year I spent kind of being a bit different to everyone else,”* even though she was referred to the PSU by her supportive manager. As their medical career impacted on their own health and vice versa, many participants reported feeling like a failure and that they should abandon their careers. For example, participant 5 stated that she was “*totally overwhelmed…because I just couldn’t cope with everything*”, and participant 6 that “*I was feeling very much like I didn’t know if I wanted to be a doctor anymore because I lost, you know, a lot of confidence in that*”. They experienced guilt when needing to take sick-leave, related to the impact this had on colleagues.

### Being on the edge: accepting help

By the time the participants accessed the PSU, many were at breaking point and their medical identity was seriously disrupted. This is illustrated by participant 2 who stated, *“I was in a position where I was either going to give up medicine, because I felt that it was negatively impacting my own health, or I was going to try and find a better way of me doing it”.*

Whilst it was often a relief finally to get support, some participants reported negative feelings associated with their initial PSU referral, which had potential to impact further on this spoiled identity; *“I kind of felt as if I was in trouble and it almost felt as if you were back at school*” (PPT 4). The referral to the PSU initially felt punitive; *“I think I felt really under surveillance*” (PPT 4). These feelings often related to the association between personal problems and medical incompetence. Most participants stressed the importance of keeping their personal problems separate from work, to avoid being judged by colleagues as not coping.

The confidentiality offered by the PSU appealed to trainees, as their colleagues did not need to know that they had accessed it. However, some remained concerned that their PSU involvement might affect their training and some were anxious about being seen in the Deanery building itself. These issues, of being under surveillance and being visible, relate to participants’ sense of threat that their covering up and coping strategies were being made visible to colleagues.

### Role of PSU in the ‘recovery’ process

Despite their initial reservations, trainees did engage with the support offered to them by PSU and found the experience “*incredibly therapeutic”* (PPT 8). However, the importance of being in the right frame of mind to access help was highlighted, as well as the need to take it at their own pace.

Analysis identified three sub-themes within the process of ‘recovery’;

(i) *Making sense of their story:* Junior doctors realised the value of being able to talk through their problems with a detached third party. In particular, taking time to process their emotions in a safe place and making sense of their story was crucial. During this process participants highlighted the importance of not being judged by PSU staff. The therapeutic the process of writing down her story was offered by one participant as both closure and also a record for the future. This was described as a “*pivotal moment*” in her recovery. Participant 8 also used this phrase when discussing how the ability to be “*totally open and honest*” about her difficulties in PSU support sessions enabled her recovery. The responses of PSU staff were crucial in validating trainees’ feelings, enabling them to view their experiences from another person’s perspective.
(ii) *Focussing on their own health:* Being supported to prioritise their own health, in order to manage difficulties, was an important part of restoration. Participants explained how reassuring it was to be told that being off work for a while could be the right thing to do as part of getting better. This is interesting as it contradicts the socialisation of trainee doctors into a culture where taking time off work is unacceptable. It may be important that trainees were able to accept this message from PSU staff when they had previously rejected it from their medical colleagues. For example, participant 6 stated she was feeling “*bad about it [sick leave] and you don’t know what everyone thinks…[the PSU staff member] was very reassuring and saying* ‘*it sounds like the right thing you’re off’*” . Several participants referred to gradually accepting that they would get to return to work more efficiently in the long run if they accepted some sick leave now.
(iii) *Understanding that they are not alone:* It was also crucial for participants to feel that they were not the only trainee who had problems. This helped them to feel more normal amongst their medical colleagues and to accept that they are human. Participant 7 exemplified this when she said “*it’s just nice knowing that I wasn’t the first trainee that had ever been through this sort of thing*”. Many participants referred to being supported and not judged shifting their notions of their traineeship being irrevocably damaged by their need for support.

### Repaired identity / Coming back from the edge

Many junior doctors were extremely concerned about their competence to continue practising medicine and some indicated that the support from PSU was essential to keeping them working as a doctor; *“they… initiated a whole lot of support that I probably couldn’t have managed without”* (PPT 1) and “*this [PSU] is the one thing that’s really essentially kept me in my job and kept me carrying on a doctor when I was so close to just giving it all up*” (PPT 8). Junior doctors expressed the sense that the PSU wanted to help get them back on track and keep them in training. PSU staff did this by providing practical support, such as liaising with other agencies on their behalf, which was particularly helpful for participants who were depressed; *“she [PSU staff member] filled it [a form] in on my behalf, because to be honest at that stage I was just a bit of a mess…and I just couldn’t do almost anything”* (PPT 8). The support that participants received helped them to feel valued as a doctor, thus assisting in repairing their spoiled identity. This strongly links to the notion of feeling normal, as trainees realised that others who had experienced difficulties had come through these.

### Different ways to be a doctor

Through their interactions with the PSU, participants were supported to explore different options to continue working medically, or to ‘do things differently’, such as working part time. In contrast with their previous concerns about overloading colleagues with extra work, some participants came to understand that they needed reduced hours, or amended duties, in order to survive as a doctor. Knowing that it would be for a short amount of time, leading to positive work outcomes, helped to reduce associated feelings of guilt. Such temporary support meant that trainees were able to continue working, which was often helpful in demonstrating to them that were capable of continuing to be a doctor. Participant 2 exemplified this stating *“the overwhelming feel [during PSU support] is that actually you can do things in different ways and that doesn’t make you any less of a person”*.

### Breaking the cycle

Analysis indicated that change was needed to break the cycle of shame about accepting help to continue practising medicine. This was both at an individual level and at a broader cultural level, within medicine. At an individual level, trainees highlighted that knowing someone was actively interested in their ongoing well-being was important and provided a safety net in case they should need future support. For example, participant 8 discussed how “*I just knew that I had someone that I could go back to, it was then the continued support after that*”. Participants suggested that in order to challenge broader cultural expectations of doctors, it could be helpful to hear from other junior doctors who have had similar experiences and who were now on the ‘other side’. Mentoring, or peer support was suggested. One participant felt that there were a number of ‘messages and learning points’ from her experience and wished to share these to prevent others’ suffering. It was also suggested that a clear protocol for future similar events including earlier referral to PSU would be helpful for other doctors who may experience difficulties.

## Discussion

Our findings show that trainee doctors are highly motivated to preserve their identity as a doctor who copes well with the demands of training. They do not wish to be seen as different from the normal medical identity that doctors perform well at work even when stressed or unwell. We interpreted our findings in the light of the theoretical framework of stigma, first suggested by Goffman [32]. He defined stigma as a social response to deviance, i.e. ‘normal’ members of a society label those who have transgressed social norms. Goffman’s empirical and theoretical work showed that stigma confers a spoiled identity on recipients, who may internalise it. However, they are not passive recipients but manage stigma by minimising its significance (termed ‘covering’) or concealing it (‘passing’). Stigmatised individuals may come together and promote acceptance of their state. Alternatively, they may simply adopt identities accepted by ‘normals’.

Our findings reveal that junior doctors often wait until they are right ‘on the edge’ before accessing the PSU, but they did internalise the concept of stigma, feeling uncomfortable because they began to perceive themselves as different from colleagues, who all appear to be coping. Trainees wanted to ‘pass’ as a resilient, stoical doctor rather than being seen as someone who cannot cope. This reluctance to take time out to ‘recover’ might also be explained by concerns that colleagues would have to suffer extra workloads on their behalf. Whilst this is understandable, it contributes towards perceptions of stigma and that their medical identity is spoiled [13] as happens to patients’ identities of being a competent worker when they take time off [33]. This also relates to other research on stigma showing that some conditions can be viewed as ontologically offensive since they disrupt social discourses [34]. Here, our analysis shows trainees were concerned about disrupting the socially imbued notion that doctors do not create work for colleagues by taking time off. Waiting until one is ‘on the edge’ also links to previous empirical work suggesting that the demands of doctoring can be difficult for some to bear [6].

It is notable that merely accessing the PSU initially exacerbated the feelings of spoiled medical identify for some participants. For example, some junior doctors were so worried about confidentiality that simply being seen in the Deanery building concerned them. This reflects Goffman’s notion of people covering up stigma and being worried if such cover is removed. This concern about visibility is supported by concerns raised by participants in other studies, particularly regarding mental health [35]. Participants coped with these concerns by trying to minimise the likelihood that others would know. Concern about being visibly seen to accept help also may be related to the feeling of being under surveillance. Our findings suggest that the initial act of seeking help changed the surveillance from an accepted educational mechanism into some kind of stigmatising process. This is worrying, as it is unlikely to promote active help-seeking by those who need support.

However, once junior doctors experienced how helpful the PSU was, they were much less concerned about its visibility and the role in their lives. Crucially, the support trainees received enabled them to repair their self-identities, as they recalibrated their ideas of what a normal trainee is, realising that others who experienced difficulties had got through them. Analysis showed that once participants felt better, they wanted to pass on what they had learned about their recovery from difficulties to other trainees. This is similar to Goffman’s notion that individuals who have felt stigmatised can lobby for change in others’ views, to alter what is considered as normal. In this sense, the PSU itself also functions as a positive agent for change against stigma, since it was the PSU support provided that enabled trainees to start to feel better. Indeed, data show that trainees particularly valued the ongoing nature of PSU support, even once initial problems had been managed. There was a transformation from the PSU exacerbating feelings of spoiled medical identity initially, towards its ‘pivotal’ role in the repair of this identity over time. The PSU assisted with identity reparation by enabling participants to make sense of their recovery journey. Once participants realised that they were not being judged, but were being supported emotionally and practically (via active listening, realising they are not the only ones needing help, writing as therapy, and reduced hours), they were able to accept the support offered to them and return to productive work. This suggests the PSU was able to help in reducing potential attrition from work, an important finding given the societal and financial cost of losing junior doctors [21].

The provision of support services is important to support well-being among doctors, within the context of their well-documented health needs [35, 36]. However, this study suggests that junior doctors do not appear to take on board information about the PSU given to them at induction. Previous studies have shown that anxiety peaks during the induction period and may interfere with their capacity to absorb the content of their induction training [15]. Our findings further suggest that the lack of attention paid to sources of support may be because they have already absorbed underlying cultural messages around doctors being largely invincible [13, 37]. This is supported by research indicating that doctors are discouraged from acknowledging personal illness, and that these messages proliferate during training, whilst they are being socialised into the medical profession [18]. It could be argued that such socialisation is part of the “hidden curriculum”, whereby cultural beliefs and behaviours are enacted by senior medical staff and transmitted to students [38, 39]. This unwillingness to acknowledge personal illness may be learnt as early as medical school, where the fear of jeopardising academic status is a barrier to help-seeking amongst medical students [20]. There is some evidence that doctors who have been ill discuss how eventually, disclosing and experiencing illness makes them better doctors in the future through greater patient empathy [40]. However, our data did not support this as our findings show that participants were concerned not to break their identity as a doctor in training who is coping well.

Our data suggest that reminding trainee doctors at regular intervals of the existence of the PSU may be helpful in encouraging earlier intervention before trainees reach breaking point and this could reduce stigma, simply by normalising the existence of a PSU. Several participants offered to talk to doctors who were in distress, now that they felt they had been through a very difficult time and emerged intact, indeed, in some cases, with a stronger recognition that there are different ways to be a successful doctor. Our participants did not detail how such interaction with other trainees could occur. There is an established literature on medical mentoring schemes [41–43] but we did not find any studies evaluating if trainee doctors who have recently emerged from difficulties can usefully mentor other junior doctors who are currently having problems, so this may be a sensible forum to explore arising from our study. However, as participants in this study noted, it is very difficult to shift a wider cultural set of expectations about long working hours and not being ill.

### Strengths and limitations of the study

Recruitment and data collection for this study occurred before the current junior doctors’ dispute in the UK. This dispute may be adding stressors to doctors’ working lives, such as concerns about how the general public views them. However, our themes are still likely to be salient, such as how personal and professional stressors interact and how the nature of being a junior doctor makes it especially difficult to ask for help. It appears unlikely that such stressors would be reduced in the context of one’s profession being in dispute with the Government. Researching how the current macro context of being a junior doctor affects individuals’ experience of seeking help whist in training would be useful.

This study is composed of a small sample. We do not know the precise reasons for the low response rate although we suggest that stigma may play a part, as highlighted here and also by previous research [24]. As mentioned, whilst all our respondents were female, this applied to only 65% of PSU users within the study period. As stated above, we found some evidence in the general population that offering incentives mean women complete more items in telephone surveys than men [30]. It is possible that male doctors would have responded more to an anonymous questionnaire than to requests for interviews, a method which could be adopted and trialled in future studies. It is known that within the general population, more women seek medical help for psychological distress than men [31] and that within the medical population, female doctors are less likely to confide in colleagues than male [44]. This may mean that female doctors are more likely to use a source of support which does not involve direct colleagues, such as the PSU. Such evidence may explain why there is a higher percentage of women using the PSU than men. We do not know why all those who responded to our study invitation were female. This may be linked to women’s experiences of being more likely than men to have accessed help for mental distress, since they may be more used to articulating such experiences. It is possible that after male doctors access PSU support, they are less willing to revisit, or reflect on periods of personal difficulty, which might indicate their allegiance to the notion of ‘manning up’ and ‘getting on with it’. Our female sample means that we cannot extrapolate our findings to male doctors. The small sample also means that we cannot interrogate the data for other patterns, such as whether differences in specialities were important, which has been shown in other relevant contexts. For example, it has recently been argued that surgery is viewed as a more ‘macho’ specialty than others which may have implications for help-seeking [45]. However, the sample provided a useful opportunity to explore the experience of female trainees. We highlight that the perceived NHS requirements to “man up” may not be in alignment with female doctors’ notions of their personal identities, but we found that most respondents spoke in terms of being “only human” rather than a more gendered experience. Many of the themes we have presented are closely related to those in the established literature about the professional socialisation of doctors, both male and female, into notions of being invulnerable. More research into how both male and female doctors experience PSUs is needed.

This is a small sample of female junior doctors at different points in their traineeships from one Deanery. We have provided a rich data set and theoretically informed, explanatory approach to our analysis. These elements, together with our description of the Deanery’s aims and mechanisms regarding supporting its trainees, of our methodology and of our findings, should enable readers to decide whether they can compare our results to their own populations of interest [46]. Although all users of PSU were invited to participate, interviewees were self-selecting, which may reflect their wish to discuss either a very positive or negative experience of the PSU. Whilst our findings in relation to evaluating the PSU are almost entirely positive, we acknowledge that the recruitment email originally came from administrative staff at the Deanery, which may have increased potential response bias. Telephone interviews have been described as inferior to face-to-face interviews, for fostering rapport and recognising subtleties of communication [47]. However, they can also help interviewees feel comfortable when talking about sensitive topics within a medical context [48]. In our study the latter was relevant, since participants opened up about extremely sensitive events.

## Conclusions

The PSU represents one example of an important source of support for junior doctors. Those who accessed PSU support in this study progressed from almost giving up medicine to feeling that they were acceptable as doctors again. We acknowledge the small, gendered sample and have provided a theoretically informed description to enable readers to consider our findings in the light of their own circumstances. Reducing the stigma attached to help-seeking is crucial to increase ease of access to support systems (such as the PSU) and to improve the experiences of doctors who encounter challenges during their training. The theme of “breaking the cycle” suggests ways in which PSUs, or similar services, could reduce stigma. Trainees who had successfully been supported by the PSU could talk to junior doctors at induction, or could informally mentor others. However care should be taken to avoid generating anxiety among trainees by discussing potential problems at induction. Our analysis shows that none of the junior doctors anticipated their vulnerabilities until they were almost ‘on the edge’, making it even harder to seek out and activate help. This extremity suggests that it is important to increase awareness among doctors that seeking help does not reflect weakness. Indeed the culture for junior doctors reflected in our findings remains predominantly concerned with “manning up”. A shift of emphasis towards the idea that it is all right to access support services when needed would be welcomed. In Goffman’s terms, this may reduce stigma as trainees in difficulty no longer need to find ways to cover up problems and pass as ‘normal’ but find that the very act of seeking help can itself be normalised as part of continuing to be a valued and valuable trainee doctor. Our findings suggest that this shift of emphasis could enable doctors to view the act of help-seeking as a positive part of learning and development, rather than a potentially catastrophic career-defining event.

## Abbreviation

PSU: Professional Support Unit
